# Experience feedback on the management of an Ebola patient in a Guinea-based healthcare facility

**DOI:** 10.1186/2047-2994-4-S1-P2

**Published:** 2015-06-16

**Authors:** B Ndoye, OF Bangoura, AO Diallo

**Affiliations:** 1 ICAN, Senegal; 2 WHO, Guinea; 3 Ministry of Health, Guinea

## Introduction

During the Ebola virus epidemic 2014-15, the case of a health facility where two doctors were contaminated was investigated to determine the reality of facts and circumstances that led to the case happening, in order to determine the causes and contributing factors to ultimately propose corrective actions.

## Objectives

The aim is to raise awareness about the high risks of contamination in the facilities and to set up recommendations for all Guinean facilities.

## Methods

A systematic analysis of the facts observed using the Orion method with phases of information collection and validation, followed by a variation and contributing factor analysis.

## Results

The direct causes observed are mainly due to failure to comply with recommended good healthcare security practices, in particular standard and additional precautions during any healthcare. Contributing factors are mainly the lack of an infectious risk management program in general and the healthcare facility being unprepared for Ebola patient management in particular.

## Conclusion

Suggested corrective actions include local administrative, organizational and technical measures to receive suspected cases and implement recommended healthcare precautions. They also relate to national measures to support healthcare facilities.

This example is illustrative of the Guinean health system’s current difficulties facing the epidemic for the component of infection control in healthcare facilities without international support. Substantive long term work will be necessary to effect changes in the organization of healthcare practices, but in all healthcare facilities, there is an urgent need to set up a secure system to handle the suspected cases they are supposed to receive any time during the epidemic.

## Disclosure of interest

None declared.

